# Supplementary material for: Silicone Induced Granuloma of Breast Implant Capsule (SIGBIC) diagnosis: Breast Magnetic Resonance (BMR) sensitivity to detect silicone bleeding
Source: PLoS One. 2020 Jun 26;15(6):e0235050. doi: 10.1371/journal.pone.0235050 (PMC7319285; doi:10.1371/journal.pone.0235050)
Supplement: S1 File — (PDF) [file pone.0235050.s001.pdf]

ABG CONSULTORIA ESTATÍSTICA

Conselho Regional de Estatística
Registro número 099 - 6ª Região

RELATÓRIO ESTATÍSTICO

Produção Científica e Tecnológica

SAIBA MAIS ▶

www.abgconsultoria.com.br

(31) 2516-0068

Conteúdo

Conteúdo 2
Sumário de Tabelas 2
Glossário 3
Objetivo 4
Metodologia 5
Análise descritiva 6
Análise univariada 7
Análise multivariada 8
Referências 11

Sumário de Tabelas

Tabela 1 - Descriptive analysis 6
Tabela 2 - Univariate analysis 8
Tabela 3 - Multivariate analysis 9
Tabela 4 - Quality statistics of the multivariate model 10

Glossário

P-valor: É uma estatística utilizada para sintetizar o resultado de um teste de hipóteses. Formalmente, o p-valor é definido como a probabilPatient Age de se obter uma estatística de teste igual ou mais extrema que aquela observada em uma amostra, assumindo como verdadeira a hipótese nula. Como geralmente define-se o nível de significancia em 5%, uma p-valor menor que 0,05, gera evidências para rejeição da hipótese nula do teste.

D.P. – Desvio Padrão. É uma das principais medidas de dispersão dos dados. Pode ser definida como a raiz quadrada da variância. Sua medida representa o quanto os dados se afastam da média.

E.P. - Erro Padrão: O erro padrão é uma medida da precisão da média amostral. O erro padrão é obtido dividindo o desvio padrão pela raiz quadrada do tamanho da amostra.

1ª Q – 1ª Quartil: O primeiro quartil é uma medida de posição que representa que pelo menos 25% das respostas são menores que ele.

2ª Q – 2ª Quartil: O segundo quartil, também conhecido como mediana é uma medida de posição que representa que pelo menos 50% das respostas são menores que ele.

3ª Q – 3ª Quartil: O terceiro quartil é uma medida de posição que representa que pelo menos 75% das respostas são menores que ele.

I.C – 95% = Intervalo de 95% de confiança: É um intervalo estimado para um parâmetro estatístico. Em vez de estimar o parâmetro por um único valor é dado um intervalo de estimativas prováveis. Um intervalo de 95% de confiança garante que o parâmetro pontual estimado com 95% de confiança estará dentro do intervalo estimado em outras amostras da mesma população.

Objetivo

- Realizar uma análise descritiva;
- Comparar as variáveis numéricas e categóricas entre os grupos NON-SIGBIC e SIGBIC

Metodologia

O banco de dados era composto por 680 pacientes e foram coletadas variáveis de caracterização e relacionadas a SIGBIC (Silicone-Induced Granuloma of Breast Implant Capsule).

Na análise descritiva das variáveis categóricas de interesse foram utilizadas as frequências absolutas e relativas, ao passo que na descrição das variáveis numéricas foram utilizadas medidas de posição, tendência central e dispersão.

A fim de avaliar os fatores que exerciam influência sobre SIGBIC foi realizada inicialmente uma análise univariada via testes Qui-Quadrado (AGRESTI; KATERI, 2011) e teste Exato de Fisher (AGRESTI; KATERI, 2011) para o caso de variáveis categóricas e teste de Mann-Whitney (HOLLANDER; WOLFE, 1999) para o caso de variáveis numéricas. Através da análise univariada foram selecionados os potenciais preditores para a variável resposta, sendo considerado um nível de significância igual a 25%. Posteriormente, a partir das variáveis selecionadas na análise univariada, foi ajustado um modelo multivariado de Regressão Logística (AGRESTI, 2002) e neste modelo foi aplicado o método *Backward* (EFROYMSON, 1960). O método *Backward* é o procedimento de retirar, por vez, a variável de maior valor-p, repetindo o procedimento até que restem no modelo somente variáveis significativas. Para o método *Backward* foi adotado um nível de 5% de significância.

Para verificar se os modelos ajustados estavam adequados e se possuíam boa capacidade preditiva foram calculadas algumas medidas de qualidade de ajuste, sendo elas: Pseudo R^2 (NAGELKERKE, 1991), AUC (área sob a curva ROC), Sensibilidade, Especificidade, VPP, VPN e o teste de Hosmer-Lemeshow (AGRESTI; KATERI, 2011).

O software utilizado nas análises foi o R (versão 3.5.2).

Análise Descritiva

A tabela 1 apresenta a análise descritiva das variáveis do estudo. Deste modo, observa-se que:

- 30,59% da amostra teve SIGBIC e todos os pacientes tiveram Capsular Contracture.
- Os indivíduos sem Intracapsular Rupture e Extracapsular corresponderam a 94,12% e 99,41% da amostra, respectivamente.
- 18.68% tiveram water droplet "yes", 93.38% não tiveram Implant Rotation e 0.59% apresentaram Siliconoma Extracapsular "yes".
- O Lymph node categoria "yes" teve representatividade de 7.06%, enquanto o Pericapsular edema 5.74%.
- A categoria "yes" do Seroma Intracapsular correspondeu a 12.06% da amostra, ao passo que o motivo diagnóstico teve a maioria dos casos (82.35%).
- 80.44% tiveram como Type of surgery a Aesthetic, 97.50% Type of Implant categoria silicone e 71.13% o Location of Implant subglandular.
- O valor médio de Patient Age foi 42.26 anos com desvio padrão de 11.17 e o time of surgery foi 7.39 anos.

Tabela 1 - Descriptive analysis

Variables	Category	Absolute Freq.	Relative Freq.
SIGBIC	No	472	69.41%
	Yes	208	30.59%
Capsular Contracture	Yes	680	100.00%
Intracapsular Rupture	No	640	94.12%
	Yes	40	5.88%
Extracapsular Rupture	No	676	99.41%
	Yes	4	0.59%
Water droplet	No	553	81.32%
	Yes	127	18.68%
Implant Rotation	No	635	93.38%
	Yes	45	6.62%
Siliconoma Extracapsular	No	676	99.41%
	Yes	4	0.59%
Lymph node	No	632	92.94%
	Yes	48	7.06%
Pericapsular edema	No	641	94.26%
	Yes	39	5.74%
Seroma Intracapsular	No	598	87.94%
	Yes	82	12.06%
Cause	Tracking	120	17.65%
	Diagnostic	560	82.35%
Type of surgery	Aesthetic	547	80.44%
	Oncologic	133	19.56%
Type of Implant	Silicone	663	97.50%
	Saline	15	2.21%

	Double	2	0.29%
Location of Implant	Subglandular	483	71.13%
	Submuscular	196	28.87%
Patient Age (years)	Mean (Mean ± S.D.)	42.26	[31.09; 53.43]
Time of surgery (years)	Mean (Mean ± S.D.)	7.39	[1.76; 13.02]

Análise Univariada

A tabela 2 exibe a análise univariada via teste Qui-Quadrado (AGRESTI, 2002) para variáveis categóricas explicativas com frequências esperadas maiores que 5 em todas as classe e teste Exato de Fisher (AGRESTI, 2002) para os casos contrários. Para as variáveis numéricas (Patient Age e Time of surgery) foi feita a análise via teste de Mann-Whitney (HOLLANDER; WOLFE, 1999). As variáveis selecionadas na análise univariada foram Intracapsular Rupture (valor-p = 0.094), water droplet (valor-p < 0.001), Lymph node (valor-p < 0.001), Pericapsular edema (valor-p < 0.001), Seroma Intracapsular (valor-p < 0.001), motivo (valor-p < 0,001), Type of surgery (valor-p = 0.023), Type of Implant (valor-p = 0.066) e Patient Age (valor-p = 0.026), uma vez que apresentaram um valor-p inferior a 0.250. A variável Capsular Contracture não participou desta análise visto que todos os indivíduos tiveram categoria sim. Isto posto, verifica-se que:

- Houve associação significativa (valor-p < 0.001) entre a variável "water droplet" e SIGBIC, sendo que entre os indivíduos com categoria "yes" de water droplet, 51.97% tiveram SIGBIC, enquanto para a categoria "no" este número foi maior (25.68%).
- Houve associação significativa (valor-p < 0.001) entre a variável "Lymph" node e SIGBIC, à vista disso 60.42% dos indivíduos com categoria "yes" para Lymph node tiveram SIGBIC, a medida que para a categoria "no" do Lymph node, 28.32% tiveram SIGBIC.
- Houve associação significativa (valor-p < 0.001) entre a variável "Pericapsular edema" e SIGBIC, por conseguinte considerando Pericapsular edema negativo, 27.93% apresentaram SIGBIC, ao passo que Pericapsular edema positivo 74.36% tiveram SIGBIC.
- Houve associação significativa (valor-p < 0.001) entre a variável "Seroma Intracapsular" e SIGBIC, destarte 27.76% dos pacientes que não tiveram seroma apresentaram SIGBIC, enquanto que 51.22% dos pacientes que tiveram seroma apresentaram SIGBIC.
- Houve associação significativa (valor-p < 0.001) entre a variável "cause" e SIGBIC, sendo que 9.17% dos pacientes que tinham como causa "tracking" apresentaram SIGBIC, enquanto que 35.18% dos pacientes que tinham como causa "diagnostic" apresentaram SIGBIC.
- Houve associação significativa (valor-p = 0.023) entre a variável "Type of surgery" e SIGBIC, sendo que 28.52% dos pacientes que fizeram cirurgia "aesthectic' apresentaram SIGBIC, enquanto que 39.10% dos pacientes que fizeram cirurgia "oncologic" apresentaram SIGBIC.
- Houve associação significativa (valor-p = 0.026) entre a variável "Patient Age" e SIGBIC, sendo que os pacientes com SIGBIC apresentaram uma idade maior.

Tabela 2 - Univariate analysis

Variables	Category	SIGBIC = No		SIGBIC = Yes		P-value
		N	%	N	%	
Capsular Contracture	No	-	-	-	-	-
	Yes	472	69.41%	208	30.59%	
Intracapsular Rupture	No	439	68.59%	201	31.41%	0.094[1]
	Yes	33	82.50%	7	17.50%	
Extracapsular Rupture	No	468	69.23%	208	30.77%	0.319[2]
	Yes	4	100.00%	0	0.00%	
Water droplet	No	411	74.32%	142	25.68%	**<0.001[1]**
	Yes	61	48.03%	66	51.97%	
Implant Rotation	No	437	68.82%	198	31.18%	0.274[1]
	Yes	35	77.78%	10	22.22%	
Siliconoma Extracapsular	No	469	69.38%	207	30.62%	1.000[2]
	Yes	3	75.00%	1	25.00%	
Lymph node	No	453	71.68%	179	28.32%	**<0.001[1]**
	Yes	19	39.58%	29	60.42%	
Pericapsular edema	No	462	72.07%	179	27.93%	**<0.001[1]**
	Yes	10	25.64%	29	74.36%	
Seroma Intracapsular	No	432	72.24%	166	27.76%	**<0.001[1]**
	Yes	40	48.78%	42	51.22%	
Cause	Tracking	109	90.83%	11	9.17%	**<0.001[1]**
	Diagnostic	363	64.82%	197	35.18%	
Type of surgery	Aesthetic	391	71.48%	156	28.52%	**0.023[1]**
	Oncologic	81	60.90%	52	39.10%	
Type of Implant	Silicone	456	68.78%	207	31.22%	0.066[2]
	Saline	14	93.33%	1	6.67%	
	Double	2	100.00%	0	0.00%	
Location of Implant	Subglandular	342	70.81%	141	29.19%	0.290[1]
	Submuscular	130	66.33%	66	33.67%	
Patient Age	Mean (S.E.)	41.56 (0.50)		43.85 (0.82)		**0.026[3]**
Time of surgery	Mean (S.E.)	7.27 (0.24)		7.68 (0.45)		0.878[3]

[1]Teste Qui-Quadrado. [2]Teste Exato de Fisher. [3]Teste de Mann-Whitney

Análise Multivariada

A partir das variáveis selecionadas na análise univariada foi ajustado um modelo multivariado de Regressão Logística (AGRESTI, 2002) e para este modelo foi aplicado o método Backward para a seleção final das variáveis, considerando-se um nível de significância de 5%.

A Tabela 3 apresenta o modelo multivariado final para a variável SIGBIC. Isto posto, nota-se que:

- Houve efeito significativo da variável Intracapsular Rupture (valor-p = 0.028) sobre a ocorrência de SIGBIC e considerando a categoria “no” como referência para a variável Intracapsular Rupture, um indivíduo com categoria “yes” tem a chance de SIGBIC positivo reduzida em 65% [11%; 86%].

- A variável water droplet teve efeito significativo (valor-p < 0.001) sobre a ocorrência de SIGBIC, desta forma a categoria referenciada sendo "no", um indivíduo com water droplet "yes" tem a chance de ter SIGBIC multiplicada por 2.82 [1.83; 4.37].
- Houve efeito significativo da variável Lymph node (valor-p = 0.001) sobre a ocorrência de SIGBIC, logo a categoria referência sendo "no", um paciente com Lymph node "yes" tem a chance de SIGBIC multiplicada por 3.07 [1.54; 6.13].
- A variável Pericapsular edema foi significativa (valor-p < 0.001) e considerando a categoria Pericapsular edema "no" como referência, um indivíduo com Pericapsular edema "yes" tem a chance de SIGBIC multiplicada por 5.02 [2.26; 11.14].
- A variável Seroma Intracapsular foi significativa (valor-p = 0.001) sobre o efeito de SIGBIC, sendo assim um paciente com Seroma Intracapsular tem uma chance de ter SIGBIC 2.40 [1.41; 4.09] vezes maior que um paciente que não tem Seroma Intracapsular.
- A variável motivo foi significativa (valor-p < 0.001) e considerando a categoria "tracking" como referência, um indivíduo com cause "diagnostic" tem a chance de ter SIGBIC multiplicada por 5.07 [2.58;9.98].
- A variável Type of surgery foi significativa (valor-p < 0.001) e caso a categoria de referência seja Aesthetic, uma paciente com Type of surgery Oncologic tem a chance de ter SIGBIC multiplicada por 1.94 [1.20; 3.13].
- A variável Patient Age foi significativa (valor-p = 0.042) sobre o efeito de SIGBIC, por conseguinte a cada aumento de um ano na Patient Age do indivíduo, a chance de ter SIGBIC positiva aumenta 2% [0%;4%].

Tabela 3 - Multivariate analysis

Variáveis	O.R.	I.C. (95%)	Valor-p
Intracapsular Rupture = No	1.00	-	-
Intracapsular Rupture = Yes	0.35	[0.14; 0.89]	**0.028**
Water droplet = No	1.00	-	-
Water droplet = Yes	2.82	[1.83; 4.37]	**<0.001**
Lymph node = No	1.00	-	-
Lymph node = Yes	3.07	[1.54; 6.13]	**0.001**
Pericapsular edema = No	1.00	-	-
Pericapsular edema = Yes	5.02	[2.26; 11.14]	**<0.001**
Seroma Intracapsular = No	1.00	-	-
Seroma Intracapsular = Yes	2.40	[1.41; 4.09]	**0.001**
Cause = Tracking	1.00	-	-
Cause = Diagnostic	5.07	[2.58; 9.98]	**<0.001**
Type of surgery = Aesthetic	1.00	-	-
Type of surgery = Oncologic	1.94	[1.20; 3.13]	**0.006**
Patient Age	1.02	[1.00; 1.04]	**0.042**

Na Tabela 4 são apresentadas a sensibilidade (SEN), especificidade (ESP), valor preditivo positivo (VPP), valor preditivo negativo (VPN), teste de Hosmer-Lemeshow e Pseudo-R^2 na determinação de SIGBIC para o modelo final. Pode-se destacar que:

- A sensibilidade e especificidade (SPE) do modelo final foi de 74.00% e 63.80%, o que significa que esse modelo foi capaz de predizer corretamente 74.00% dos casos em que houve SIGBIC e capaz de predizer corretamente 63.80% dos casos em que não houve SIGBIC.
- A área sob a curva ROC (AUC) do modelo final foi de 0.750 e o Pseudo-R^2 foi 24.79%.
- O teste de Hosmer-Lemeshow para o modelo final indicou que o ajuste do modelo estava adequado (valor-p = 0.795).

Tabela 4 - Quality statistics of the multivariate model

SEN	SPE	PPV	NPV	Accuracy	AUC	Hosmer-Lemeshow	Pseudo-R^2
0.740	0.638	0.474	0.848	0.669	0.750	0.795	24.79%

Referências

AGRESTI, A. **Categorical analysis**. New York: John Wiley, 2002.

AGRESTI, A.; KATERI, M. **Categorical Data Analysis**. Gainesville, Florida: John Wiley, 2011. v. 45

EFROYMSON, M. A. Multiple regression analysis. In: **Mathematical methods for digital computers**. New York, N.Y.: John Wiley, 1960. p. 191–203.

HOLLANDER, M.; WOLFE, D. A. **Nonparametric Statistical Methods**. 2nd. ed. New York, N.Y.: John Wiley & Sons, 1999.

NAGELKERKE, N. J. D. A note on a general definition of the coefficient of determination. **Biometrika**, 1991.
